# Short-term association between ambient air pollution and heart rate variability: results from the population-based KORA S4 and FF4 studies

**DOI:** 10.1186/s12989-025-00645-6

**Published:** 2025-10-17

**Authors:** Yujiao Li, Susanne Breitner-Busch, Wayne E. Cascio, Siqi Zhang, Kathrin Wolf, Ina-Maria Rückert-Eheberg, Stefan Kääb, Georg Schmidt, Alexander Strom, Annette Peters, Alexandra Schneider

**Affiliations:** 1 Institute of Epidemiology, Helmholtz Zentrum München, German Research Center for Environmental Health (GmbH), Neuherberg, Germany; 2 Institute for Medical Information Processing, Biometry and Epidemiology (IBE), Faculty of Medicine, LMU Munich, Pettenkofer School of Public Health, Munich, Germany; 3 US Environmental Protection Agency, Durham, NC USA; 4 Department of Environmental Health Sciences, Yale School of Public Health, New Haven, CT USA; 5 German Research Center for Cardiovascular Disease (DZHK), Partner site Munich Heart Alliance, Munich, Germany; 6 Department of Cardiology, Medical Policlinic and University Clinic I, Munich, Germany; 7 Department of Internal Medicine I, TUM University Hospital, Technical University of Munich, Munich, Germany; 8 Institute for Clinical Diabetology, German Diabetes Center, Leibniz Center for Diabetes Research at Heinrich Heine University Düsseldorf, Düsseldorf, Germany; 9 German Center for Diabetes Research (DZD), München-Neuherberg, Germany

**Keywords:** Aging, Air pollution, Cardiovascular diseases, Heart rate variability, Cardiac autonomic function

## Abstract

**Background:**

Ambient air pollution is a major risk factor for CVDs, and a plausible mechanism is speculated to be alteration of autonomic nervous system (ANS) function. Yet, the short-term effects of air pollution on heart rate variability (HRV), a measure of ANS balance are inconsistent.

**Objective:**

This study aimed to evaluate the short-term effects of ambient PM_2.5_ and NO_2_ on cardiovascular autonomic function, and to determine vulnerable subgroups and temporal trends from repeated HRV and HR measurements over 14 years in the KORA cohort.

**Methods:**

We analyzed data from 4,032 participants in KORA S4 (1999–2001) and 1,912 in KORA FF4 (2013–2014). Air pollution data were from fixed monitoring stations, and HRV indices were derived from 5-minute ECG recordings. Generalized additive models (GAMs) and generalized additive mixed models (GAMMs) were used to assess associations.

**Results:**

In S4, each IQR increase in PM_2.5_ at the 14-day moving average was associated with a 2.32% (95% CI: − 4.41, − 0.19) decrease in SDNN and a 1.20% (95% CI: 0.16, 2.26) increase in HR. By contrast, KORA FF4 showed opposite associations, with a 0.86% (95% CI: 0.02, 1.70) increase in SDNN at lag 4 for PM_2.5_. Effect modifications by age and smoking status were observed in S4. No statistically significant associations were found in the longitudinal analysis, however, the observed trends were consistent with the effects identified in S4.

**Conclusion:**

Short-term exposure to PM_2.5_ and NO_2_ impacts cardiac autonomic function, with varying effects across study waves due to aging, smoking, medication, and lower pollution levels. Even at low ambient concentrations, these exposures impaired autonomic function via inflammation and oxidative stress, underscoring the importance of stringent air quality standards and lifestyle interventions in reducing cardiovascular risk.

**Supplementary Information:**

The online version contains supplementary material available at 10.1186/s12989-025-00645-6.

## Introduction

Ambient air pollution is recognized as the leading global environmental risk factor for morbidity and mortality [1]. According to the World Health Organization (WHO) [2], it ranks as the fourth leading cause of premature mortality globally, with an estimated 4.2 million deaths per year attributable to ambient air pollution. The vast majority of the global population (99%) is exposed to pollution levels that exceed WHO guideline thresholds [3]. Cardiovascular diseases (CVDs) are the leading cause of death worldwide, accounting for 20.5 million deaths in 2021, with approximately 80% of these occurring in low- and middle-income countries [4]. The WHO identifies air pollution as a major environmental risk factor for CVDs, underscoring the critical link between environmental quality and population health.

Cardiovascular autonomic function, involving both sympathetic and parasympathetic branches of the autonomic nervous system (ANS), modulates heart rhythm, vascular resistance, and contractility, and contributes to CVD progression. Heart rate variability (HRV), a well-established, non-invasive technique for assessing ANS function, has proven valuable in identifying individuals at elevated risk for cardiac events and mortality [5–8]. HRV is typically assessed using time- and frequency-domain indices. Time-domain indices include the standard deviation of normal-to-normal intervals (SDNN), the root mean square of successive RR interval differences (RMSSD), and the percentage of successive RR intervals differing by more than 50 ms (pNN50). Frequency-domain indices include low frequency (LF), high frequency (HF), and the LF/HF ratio. These indices reflect the balance between sympathetic and parasympathetic activity. Reduced HRV is a significant predictor of adverse outcomes in patients with myocardial infarction and heart failure [9].

Growing evidence suggests that air pollution, particularly particulate matter (PM) and nitrogen dioxide (NO_2_), adversely affects HRV and heart rate (HR) [10, 11]. Many studies have explored these associations in specific patient groups, such as those undergoing cardiac catheterization or with diabetes or impaired glucose tolerance. However, findings remain inconsistent, especially regarding PM_2.5_ effects on HRV indices at different lag times [12–14].

Given the limited evidence in general population cohorts, this study comprehensively examined short-term associations between exposure to PM_2.5_, NO_2_, and HRV indices and HR through cross-sectional and longitudinal analyses within two waves of the population-based KORA study, conducted 14 years apart. The 14-year interval also allowed us to consider the role of aging in modifying the associations. Unlike prior studies limited to PM_2.5_ or a narrow set of HRV indices, this study comprehensively evaluated PM_2.5_ and NO_2_ in relation to multiple HRV parameters and HR to better characterize the impact of short-term air pollution exposure on cardiovascular autonomic function.

## Methods

### Study population

In this study, we analyzed data from the Cooperative Health Research in the Region of Augsburg (KORA) survey 4 (S4) study (October 25, 1999 to April 28, 2001) and its second follow-up FF4 (June 3, 2013 to September 27, 2014) (https://www.helmholtz-munich.de/en/epi/cohort/kora). The research was conducted in Augsburg, a city in Southern Germany, along with two adjacent districts [15]. More details, including data collection and standardized sampling method, can be found elsewhere [15, 16]. KORA S4 included 4,261 German citizens aged between 25 and 74 years, and KORA FF4 examined 2,279 participants. ECG data were available for 4,032 individuals in S4 and 1,912 in FF4, with 1,843 participants having measurements in both study waves. Participants were invited to the KORA study center in Augsburg, Germany, where they completed a computer-assisted personal interview, a self-administered questionnaire, physical examinations, and electrocardiographic measurements during each visit.

The KORA study was approved by the ethics committee of the Bavarian Chamber of Physicians (Munich, Germany). All participants gave written informed consent.

### Covariates

Continuous body mass index (BMI) was divided into two categories: non-obesity (≤30 kg/m^2^) and obesity (>30 kg/m^2^). Occupational status was classified as employed for participants working, self-employed, or in training (more likely to be active outside of the home), and as unemployed/retired for those who were unemployed, homemakers, or retired (more likely to be active inside the home). Educational levels were classified into primary school, high school, and college [17]. Smoking status was divided into former smoker, smoker (regular, occasional), and never smoker. Alcohol consumption was classified into two levels: yes (>0 g/day) or no (0 g/day). Physical activity was categorized based on the time spent on physical exercise into low (no or almost no physical exercise), medium (about one hour per week), and high (more than two hours per week). CVD was defined as having one or more of the following conditions: hypertension, stroke, or MI [18]. Medication use was defined based on whether the participant was taking at least one type of medication related to the treatment of CVD conditions: aspirin; beta-adrenergic receptor blockers (beta-blockers); angiotensin-converting enzyme inhibitors (ACE inhibitors); diuretics; angiotensin receptor antagonists; calcium channel antagonists (calcium channel blockers); or other antihypertensives. Seasons were defined as spring: March–May; summer: June–August; autumn: September–November; winter: December–February. We also classified the seasons into a binary variable: April–September as the warm and October–March as the cold season.

### ECG measurement

In S4, all participants received standardized 12-lead ECG recordings (Hörmann Bioset 9000) after a 10-minute rest period in the supine position. Rigorous quality control procedures were followed to ensure signal quality, patient conditioning, and accurate electrode placement. The chest leads were routinely positioned using the Dalhousie University Square (DAL Square) to mark the electrode locations [19]. For this study, ECGs were filtered for quality and analyzed using the automated Hannover ECG System, as previously reported [20–22].

The HRV indices were calculated from 5-minute ECG recordings. Specifically, HR time series (tachogram) were extracted from these recordings as RR intervals. These were visually inspected for correctness and any abnormalities, including arrhythmia, ectopic beats, and other artifacts. Subsequently, NN interval time series were generated by applying an adaptive filter to the tachograms. This filter detected and replaced ectopic beats and artifacts with interpolated ‘normal’ beats. The detailed MATLAB routine used for this process has been described [23].

In accordance with the Task Force of the European Society of Cardiology and the North American Society of Pacing and Electrophysiology [24], several standard HRV indices were calculated from these 5-minute recordings. Time-domain HRV indices, including SDNN, RMSSD, and pNN50, as well as frequency-domain indices, including HF, LF, and LF/HF ratio, were computed following Task Force guidelines [24] and methods described by Voss et al. [25]. The methods used for HRV indices calculation are discussed in more detail in Voss et al. [26].

In FF4, ECG data collection mirrored the stringent procedures established in S4, with the only distinction being the ECG system utilized. Participants underwent a standardized procedure involving a 5-minute resting ECG, employing the ECGpro-system (Fa. AMEDTEC). Following the collection, all 5-minute ECG recordings were reviewed by a cardiologist, ensuring consistent and accurate assessment across the study. This adherence to rigorous quality control measures ensured high reliability and comparability of the data.

### Exposure assessment

Hourly data on air pollutants, specifically PM_2.5_ and NO_2_, along with meteorological parameters such as air temperature and relative humidity, were obtained from fixed monitoring sites [27]. Given the varying operational periods of the monitoring stations, daily time series of air pollutant concentrations were generated by integrating exposure data from different stations across specific timeframes. To ensure consistency in the time series for each pollutant, the station with the longest period of available data was selected as the primary source. Missing data points were addressed by performing linear regression analyses between the primary station and other nearby stations, choosing the monitor with the highest coefficient of determination (R^2^) to estimate missing values.

For PM_2.5_, daily average concentrations from 2004 to 2016 were acquired from the aerosol monitoring station (FH), situated approximately 1 km southeast of Augsburg’s city center and about 100 m from a major road. Established in 2004, this station serves as a representative site for the urban background of Augsburg [28]. Moreover, an urban background station was operated by the Bavarian Environment Agency (LfU), situated about 4 km south of the city center. To address missing data prior to February 1 st, 2001, PM_2.5_ values were estimated by downscaling PM_10_ concentrations using a factor of 0.68, as described previously [28]. Missing data at the primary FH station from 2004 to 2016 were supplemented by applying linear regression with data from either the LfU or BP stations, selecting the one with the higher R^2^ value for each year.

For NO_2_, daily concentrations from 1999 to 2014 were obtained from the BP station, with missing data estimated using linear regression with data from the LfU station. Meteorological data were independently collected from the LfU station, with any missing filled using corresponding data from the Haunstetten monitoring station (HAU), located 7 km south of Augsburg’s city center [27, 29].

### Statistical analysis

We excluded individuals with missing ECG data (*N* = 175) or any covariates (*N* = 34), resulting in a final sample of 4,032 participants in S4 and 1,912 in FF4.

The characteristics of the study population are presented as means and standard deviations (SD) for continuous variables and as frequencies and percentages for categorical variables.

We applied two study wave-specific generalized additive models (GAMs) and generalized additive mixed models (GAMMs) to explore cross-sectional and longitudinal associations of air pollution with assessed HR, SDNN, RMSSD, pNN50, HF, LF, and LF/HF ratios in S4 and FF4. We naturally log-transformed all outcome values to improve their alignment with the normal distributions of residuals [30]. For each pollutant, we considered two distinct exposure windows to assess both short-term and cumulative short-term effects. We investigated the short-term effects of air pollution on the concurrent day (lag0) and for single-day lags from one to four days (lag1–lag4) before the examination day. Cumulative short-term effects were considered using 2-day, 5-day, 7-day, and 14-day moving averages from the examination day. We also calculated corresponding exposure windows for air temperature and relative humidity. Covariates incorporated into our models were selected based on previous literature [31, 32]. Model 1 was adjusted for age, sex, the day of the week, season, time trend, temperature, and relative humidity. To handle non-linearity in the associations among time trends, temperature, and relative humidity, we employed regression splines. We allocated three degrees of freedom to the splines for temperature and relative humidity. Additionally, we assigned four degrees of freedom per year for each study wave to the spline modeling the time trend. Model 2 additionally included BMI, educational levels, occupational status, physical activity, smoking status, and alcohol consumption. In model 3, we further added CVD and reported medication use. The effect estimates are presented as percentage changes in the geometric mean (GM) of outcomes, with corresponding 95% confidence intervals (95% CI), for each interquartile range (IQR) increase in PM_2.5_ and NO_2_.

To investigate effect modification and to identify potentially susceptible subgroups to the impacts of PM_2.5_ and NO_2_, we included interaction terms between air pollutants and individual characteristics in model 3. The examined modifiers were age (< 65 years vs. ≥ 65 years), sex (male vs. female), obesity (BMI ≥ 30 kg/m^2^ vs. < 30 kg/m^2^), smoking status (current/former vs. never smoker), alcohol consumption (yes vs. no), educational attainment (primary school vs. high school vs. college), physical activity (low vs. medium vs. high), occupation (employed vs. not employed), season (cold vs. warm), CVD (yes vs. no), diabetes (yes vs. no), beta-blocker (yes vs. no), and cardiovascular medication use (yes vs. no).

To explore potential secondary effect modifications of air pollution on ECG parameters, we stratified the data into four groups based on combinations of sex and age. Each group was analyzed separately using model 3 to assess whether age and sex jointly influenced the observed associations.

We conducted multiple sensitivity analyses to evaluate the stability and reliability of our findings. First, we conducted two-pollutant models by concurrently including PM_2.5_ and NO_2_, as they exhibited low correlations (*r* < 0.7). Second, we only analyzed participants with two ECG measurements in both S4 and FF4 (1843 participants). Third, we excluded participants treated with beta-blockers, which could slow HR and reduce sympathetic nervous system activity. Fourth, we excluded participants with CVD. Fifth, we excluded outliers of HRV data, including HR, SDNN, RMSSD, pNN50, HF, LF, and LF/HF ratios, defined as natural log-transformed values less than the 25th percentile – 3 × SD or more than the 75th percentile + 3 × SD. Sixth, inverse probability weighting (IPW) was employed to mitigate potential selection bias arising from loss to follow-up, which is a method that accounts for potential bias from loss to follow-up by weighting individuals according to the inverse probability of being included [33]. All statistical analyses were conducted using R (version 4.3.0), with a significance level set at a two-sided p-value of less than 0.05.

## Result

### Characteristics of study participants

Our main analysis involved 4,032 participants from KORA S4 and 1,912 participants from KORA FF4. Table 1 presents the characteristics of participants by study wave. In KORA S4, the average age was 49.10 years, with 48.96% of participants being male and 41.25% identified as never smokers. By contrast, KORA FF4 had an average age of 58.64 years, with 46.23% of participants being male and 41.79% categorized as never smokers. The average BMI was 27.18 kg/m² for S4 participants and 27.62 kg/m² for FF4 participants. Participants in S4 were more likely to be current smokers and physically inactive compared to FF4. Additionally, the prevalence of diabetes and the use of beta-blockers and CVD medications were higher in FF4. In addition, among participants in S4, those with follow-up data were younger than individuals lost to follow-up in FF4. Participants in S4 with follow-up data also had lower rates of smoking, cardiovascular disease, and beta-blocker use, but a higher prevalence of high-level physical activity compared to those without follow-up data (Table S2).

**Table 1:** Descriptive statistics of participant characteristics at both examinations

	Mean (SD) or number (%)		*P* value*
	S4 (*N* = 4,032)	FF4 (*N* = 1,912)	
Age (years)	49.10 (13.85)	58.64 (11.74)	< 0.001
Sex (male)	1974 (48.96)	884 (46.23)	0.053
BMI (kg/m^2^)	27.18 (4.71)	27.62 (4.90)	0.001
Obesity (% yes)	939 (23.28)	515 (26.94)	0.003
Smoking status (% yes)			< 0.001
Current smoker	1048 (25.99)	313 (16.37)	
Former smoker	1321 (32.76)	800 (41.84)	
Never smoker	1663 (41.25)	799 (41.79)	
Alcohol status (% yes)	2931 (72.69)	1390 (72.70)	1.000
Physical activity*			< 0.001
Low	1364 (33.83)	517 (27.04)	
Medium	1844 (45.73)	894 (46.76)	
High	824 (20.44)	501 (26.20)	
Educational attainment			< 0.001
College	919 (22.79)	496 (25.94)	
High school	949 (23.54)	514 (26.88)	
Primary school	2164 (53.67)	902 (47.18)	
Occupational status (% yes)	2262 (56.10)	1045 (54.65)	0.307
Diabetes (% yes)	153 (3.79)	165 (8.62)	< 0.001
MI (% yes)	80 (1.98)	50 (2.62)	0.145
Stroke (% yes)	48 (1.19)	35 (1.83)	0.065
Hypertension (% yes)	1476 (36.61)	685 (35.83)	0.578
CVD (% yes)	1501 (37.23)	696 (36.40)	< 0.001
Intake of beta-blocker (% yes)	426 (10.57)	334 (17.47)	< 0.001
Intake of medication (% yes)	1008 (25.00)	703 (36.77)	< 0.001
Season			< 0.001
Spring	1481 (36.73)	415 (21.71)	
Summer	732 (18.15)	614 (32.11)	
Autumn	591 (14.66)	521 (27.25)	
Winter	1228 (30.46)	362 (18.93)	
Season (% cold)	2709 (67.19)	792 (41.42)	< 0.001

*BMI* body mass index, *CVD* participant has hypertension or stroke or MI, *FF4* second follow-up examination of KORA S4, *KORA* Cooperative Health Research in the Region of Augsburg, *MI* myocardial infarction, *SD* standard deviation, *S4* fourth cross-sectional health survey of the KORA cohort

* *P* value was estimated by t-test (continuous variables) or Chi-squared test (categorical variables). *Physical activity* was categorized based on the time spent on physical exercise into low (no or almost no physical exercise), medium (about one hour per week), and high (more than two hours per week)

### ECG parameters

In both study waves, we observed a strong correlation of SDNN with RMSSD, pNN50, and LF (see Tables 2 and 3). RMSSD also demonstrated a robust correlation with pNN50 and HF. The correlations among other ECG parameters were moderate to weak.

### Characteristics of air pollution

The average urban background concentrations of PM_2.5_ and NO_2_ in KORA S4 were higher than in KORA FF4 (see Table 4). However, the average daily concentrations of both pollutants exceeded the WHO air quality guideline levels of 5 µg/m³ for PM_2.5_ and 10 µg/m³ for NO_2_. PM_2.5_ concentrations were positively correlated with NO_2_ concentrations.

**Table 2:** Descriptive statistics and spearman correlation coefficients of ECG parameters in KORA S4

S4	Mean ± SD		Min	25%	Median	75%	Max	IQR	Correlation coefficients						
	Geometric	Arithmetic							HR	SDNN	RMSSD	pNN50	HF	LF	
HR (bpm)	64.2 ± 1.2	65.1 ± 10.7	36.0	58.0	64.0	71.0	133.0	13.0	1.00						
SDNN (ms)	34.7 ± 1.7	39.3 ± 22.3	3.9	25.4	34.8	47.9	298.8	22.5	−0.42	1.00					
RMSSD (ms)	23.9 ± 1.9	30.0 ± 23.1	2.3	15.2	24.1	37.4	316.4	22.2	−0.43	0.89	1.00				
pNN50 (%)	0.1 ± 4.3	0.1 ± 0.2	0.0	0.0	0.0	0.2	0.9	0.2	−0.42	0.78	0.92	1.00			
HF (ms²)	34.0 ± 4.0	85.9 ± 209.0	0.1	13.7	35.0	89.3	7134.5	75.6	−0.21	0.68	0.73	0.61	1.00		
LF (ms²)	66.6 ± 3.4	139.0 ± 286.9	0.4	30.5	67.4	150.3	7303.5	119.8	−0.22	0.74	0.67	0.50	0.65	1.00	
LF/HF	2.0 ± 2.6	3.0 ± 3.4	0.1	1.0	2.0	3.7	50.6	2.7	0.14	−0.08	−0.26	−0.26	−0.17	0.07	

*IQR* interquartile range, *LF* low frequency power (0.04–0.15 Hz), *LF/HF* low frequency to high frequency ratio, *Max* maximum, *Min* minimum, *HF* high frequency power (0.15–0.40 Hz), *HR* heart rate, *pNN50* percentage of NN intervals longer than 50 milliseconds, *RMSSD* root mean square of successive differences, *SD* standard deviation, *SDNN* standard deviation of normal-to-normal beats, *25%* the 25th percentile, *75%* the 75th percentile

**Table 3:** Descriptive statistics and spearman correlation coefficients of ECG parameters in KORA FF4

FF4	Mean ± SD		Min	25%	Median	75%	Max	IQR	Correlation coefficients					
	Geometric	Arithmetic							HR	SDNN	RMSSD	pNN50	HF	LF
HR (bpm)	64.5 ± 1.2	64.2 ± 9.8	37.0	58.0	63.0	70.0	123.0	12.0	1.00					
SDNN (ms)	30.9 ± 1.6	34.3 ± 16.1	3.6	22.9	31.5	42.7	127.2	19.8	−0.41	1.00				
RMSSD (ms)	18.7 ± 1.8	22.2 ± 13.6	2.3	12.8	19.1	27.8	104.0	15.0	−0.48	0.82	1.00			
pNN50 (%)	0.1 ± 3.8	0.1 ± 0.1	0.0	0.0	0.0	0.1	0.7	0.1	−0.38	0.72	0.93	1.00		
HF (ms²)	21.9 ± 3.5	45.0 ± 63.9	0.3	9.6	22.9	51.4	624.3	41.8	−0.28	0.69	0.85	0.86	1.00	
LF (ms²)	48.3 ± 3.2	91.3 ± 137.3	0.1	23.3	50.7	105.7	2064.1	82.4	−0.21	0.70	0.62	0.55	0.54	1.00
LF/HF	2.2 ± 2.5	3.3 ± 3.3	0.1	1.2	2.2	4.2	32.7	3.0	0.13	−0.00	−0.24	−0.19	−0.26	0.19

*IQR* interquartile range, *LF* low frequency power (0.04–0.15 Hz), *LF/HF* low frequency to high frequency ratio, *Max* maximum, *Min* minimum, *HF* high frequency power (0.15–0.40 Hz), *HR* heart rate, *pNN50* percentage of NN intervals longer than 50 milliseconds, *RMSSD* root mean square of successive differences, *SD* standard deviation, *SDNN* standard deviation of normal-to-normal beats, *25%* the 25th percentile, *75%* the 75th percentile

**Table 4:** Descriptive statistics and spearman correlation coefficients of daily levels of air pollution and meteorological parameters

	Descriptive statistics				Correlation coefficients		
	Mean ± SD		Range (min, max)	IQR	PM_2.5_	NO_2_	T
S4 (25.10.1999–28.04.2001)							
PM_2.5_ (µg/m^3^)	15.4 ± 8.2		3.0, 63.2	9.3	1.00		
NO_2_ (µg/m^3^)	36.5 ± 11.6		14.0, 79.5	14.1	0.58	1.00	
Temperature (T) (℃)	8.6 ± 7.2		−12.7, 25.9	10.9	0.02	−0.08	1.00
Relative humidity (RH) (%)	78.9 ± 12.3		40.6, 96.0	18.4	−0.02	−0.11	−0.52
FF4 (03.06.2013–27.09.2014)							
PM_2.5_ (µg/m^3^)	10.8 ± 7.8		1.3, 55.6	8.0	1.00		
NO_2_ (µg/m^3^)	27.5 ± 11.3		6.4, 74.9	15.1	0.58	1.00	
Temperature (T) (℃)	12.8 ± 7.3		−4.4, 29.2	12.1	−0.21	−0.18	1.00
Relative humidity (RH) (%)	72.9 ± 12.0		49.1, 96.7	19.7	0.02	−0.12	−0.62

*IQR* interquartile range, *Max* maximum, *Min* minimum, *NO*_*2*_ nitrogen dioxide, *PM*_*2.5*_ particulate matter 2.5 μm in aerodynamic diameter, *RH* relative humidity, *SD* standard deviation, *T* temperature

### Association between ECG parameters and short-term air pollution in different time windows

**Fig. 1:**
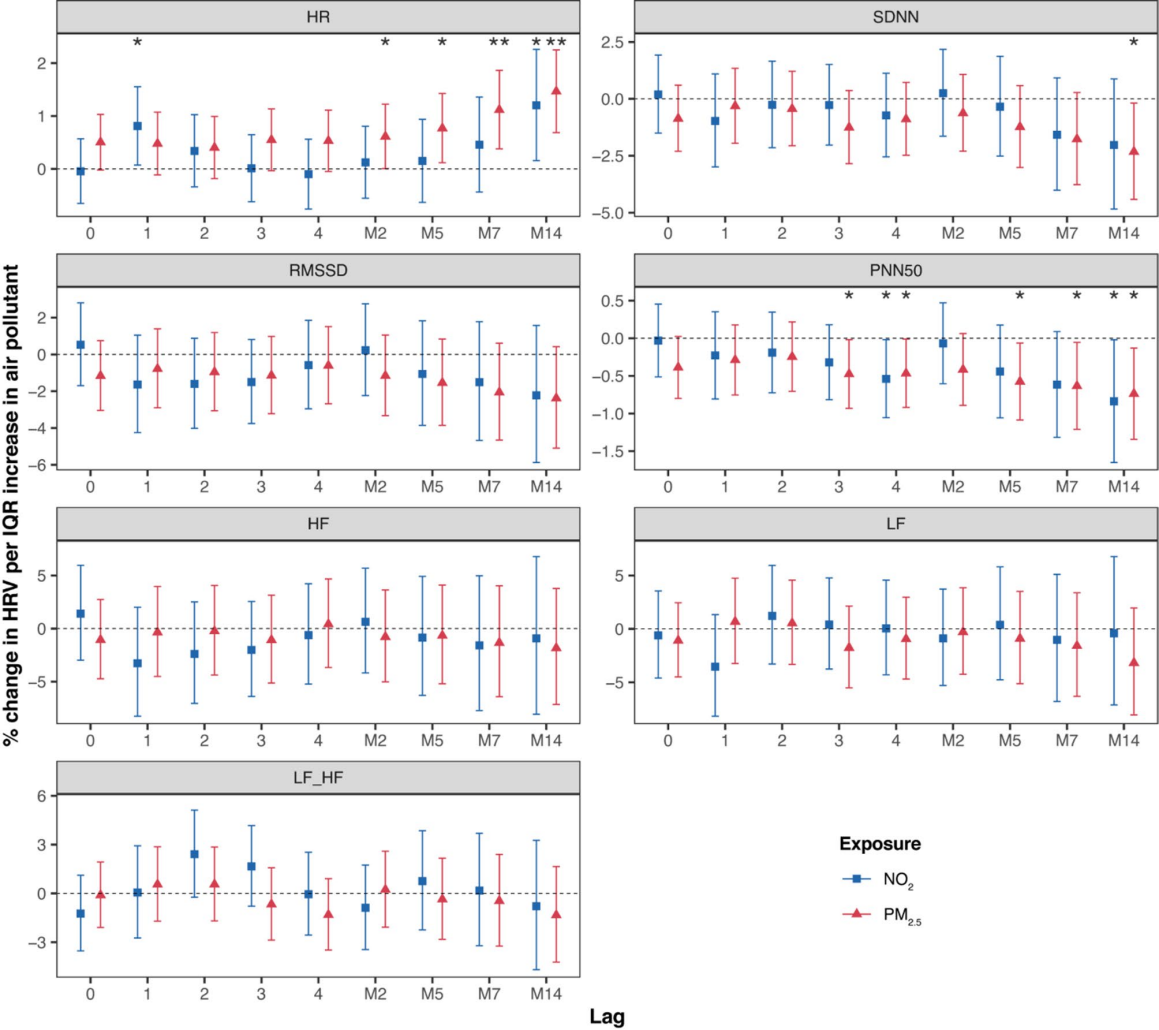
Percent change (95% CI) of the geometric mean of ECG parameters per interquartile range increase in PM_2.5_ and NO_2_ in KORA S4. *CI* confidence interval, *HF* high frequency power (0.15–0.40 Hz), *HR* heart rate, *IQR* interquartile range, *LF* low frequency power (0.04–0.15 Hz), *LF_HF* low frequency to high frequency ratio, *M2* 2-day moving average, *M5* 5-day moving average, *M7* 7-day moving average, *M14* 14-day moving average, *NO*_*2*_ nitrogen dioxide, *pNN50* percentage of NN intervals longer than 50 milliseconds, *PM*_*2.5*_ particulate matter ≤ 2.5 μm in aerodynamic diameter, *RMSSD* root mean square of successive differences, *SDNN* standard deviation of normal-to-normal beats. *p-value < 0.05; **p-value < 0.01

In the S4 analysis, we observed an increase in HR associated with each IQR increase in PM_2.5_ concentrations at the 2-day (0.61%, 95% CI: 0.01, 1.22), 5-day (0.77%, 95% CI: 0.12, 1.43), 7-day (1.12%, 95% CI: 0.38, 1.86), and 14-day (1.47%, 95% CI: 0.69, 2.25) moving averages. Similarly, HR increased with each IQR increase in NO_2_ at lag 1 (0.81%, 95% CI: 0.07, 1.55) and the 14-day moving average (1.20%, 95% CI: 0.16, 2.26) (Fig. 1).

In addition, we found significant decreases in SDNN associated with elevated PM_2.5_ concentrations at the 14-day moving average (−2.32%, 95% CI: −4.41, −0.19). For pNN50, PM_2.5_ showed the strongest cumulative effect at the 14-day moving average (0.74%, 95% CI: −1.34, −0.13) and decreases were observed with NO_2_ at lag 4 (−0.54%, 95% CI: −1.06, −0.02) and the 14-day moving average (−0.84%, 95% CI: −1.65, −0.02).

**Fig. 2:**
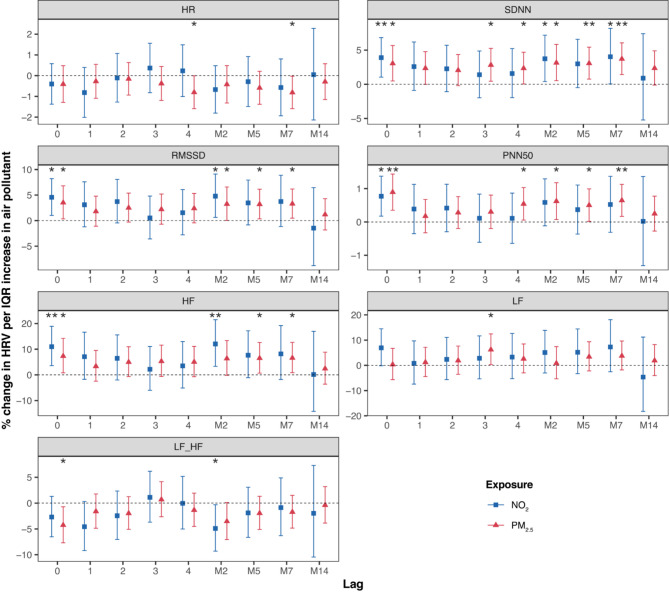
Percent change (95% CI) of the geometric mean of ECG parameters per interquartile range increase in PM_2.5_ and NO_2_ in KORA FF4. *CI* confidence interval, *HF* high frequency power (0.15–0.40 Hz), *HR* heart rate, *IQR* interquartile range, *LF* low frequency power (0.04–0.15 Hz), *LF_HF* low frequency to high frequency ratio, *M2* 2-day moving average, *M5* 5-day moving average, *M7* 7-day moving average, *M14* 14-day moving average, *NO*_*2*_ nitrogen dioxide, *pNN50* percentage of NN intervals longer than 50 milliseconds, *PM*_*2.5*_ particulate matter ≤ 2.5μm in aerodynamic diameter, *RMSSD* root mean square of successive differences, *SDNN* standard deviation of normal-to-normal beats. *p-value < 0.05; **p-value < 0.01

In contrast to the results observed in the S4 cohort, the FF4 cohort exhibited the opposite association patterns. We observed a decrease in HR associated with PM_2.5_ at lag 4 and the 7-day moving average. Positive associations with SDNN, pNN50, RMSSD, HF, and LF were observed for both PM_2.5_ and NO_2_. Notably, significant negative associations with the LF/HF ratio were observed for PM_2.5_ at lag 0 and NO_2_ at the 2-day moving average (Fig. 2).

In longitudinal analyses restricted to participants with two ECG measurements, no associations reached statistical significance. However, the overall trend aligned directionally with the S4 results (Figure S1).

### Effect modification

**Fig. 3:**
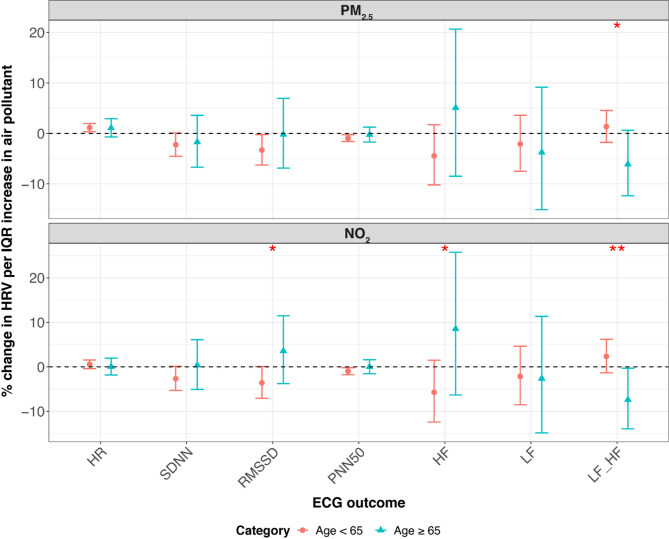
Percent change (95% CI) of the geometric mean of ECG parameters per IQR increase in PM_2.5_ and NO_2_ modified by age in KORA S4 at lag M7. *CI* confidence interval, *HF* high frequency power (0.15–0.40 Hz), *HR* heart rate, *IQR* interquartile range, *LF* low frequency power (0.04–0.15 Hz), *LF_HF* low frequency to high frequency ratio, *M2* 2-day moving average, *M5* 5-day moving average, *M7* 7-day moving average, *M14* 14-day moving average, *NO*_*2*_ nitrogen dioxide, *pNN50* percentage of NN intervals longer than 50 milliseconds, *PM*_*2.5*_ particulate matter ≤ 2.5 μm in aerodynamic diameter, *RMSSD* root mean square of successive differences, *SDNN* standard deviation of normal-to-normal beats. *p-value < 0.05; **p-value < 0.01

**Fig. 4:**
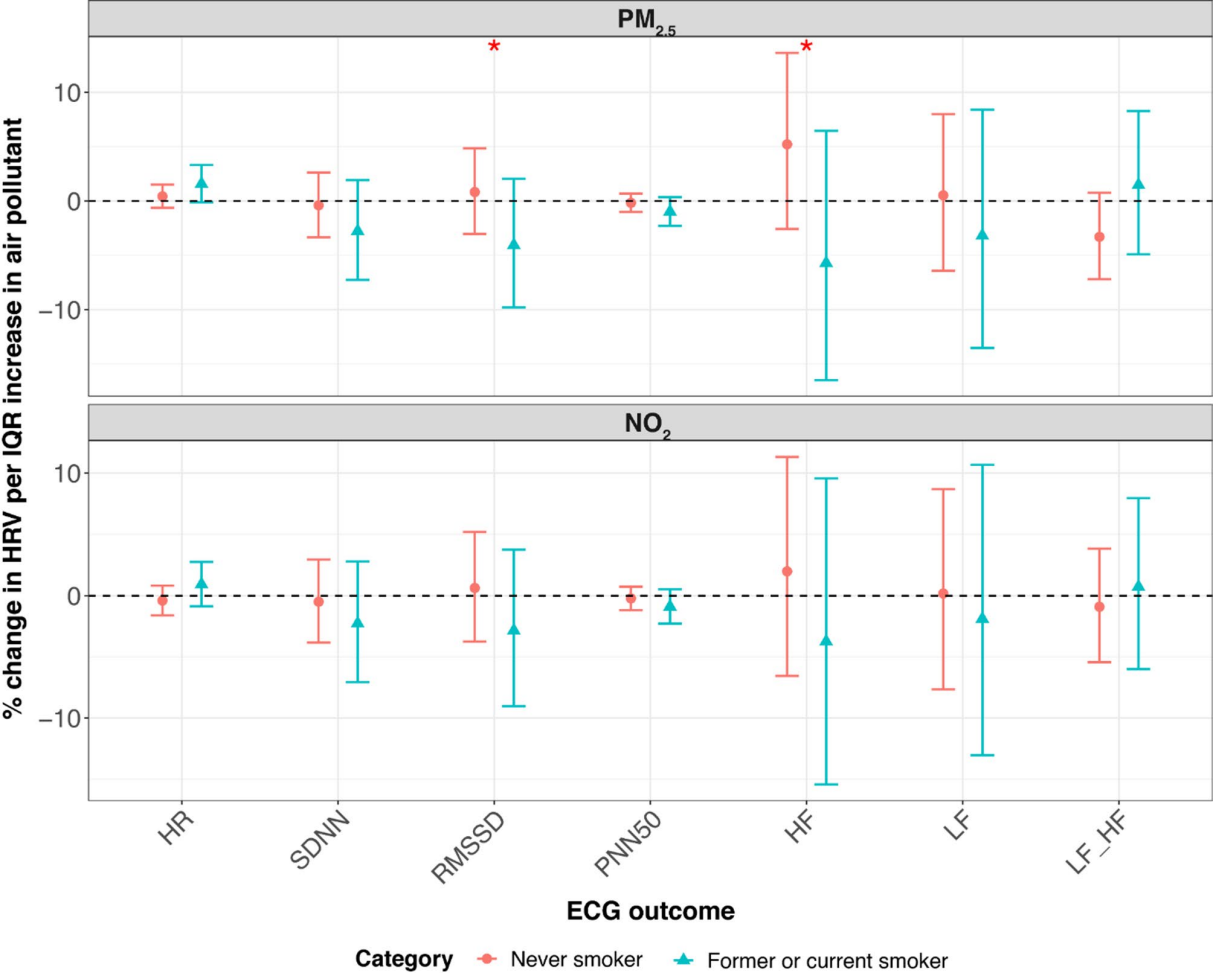
Percent change (95% CI) of the geometric mean of ECG parameters per IQR increase in PM_2.5_ and NO_2_ modified by smoking status in KORA S4 at lag M7. *CI* confidence interval, *HF* high frequency power (0.15–0.40 Hz), *HR* heart rate, *IQR* interquartile range, *LF* low frequency power (0.04–0.15 Hz), *LF_HF* low frequency to high frequency ratio, *M2* 2-day moving average, *M5* 5-day moving average, *M7* 7-day moving average, *M14* 14-day moving average, *NO*_*2*_ nitrogen dioxide, *pNN50* percentage of NN intervals longer than 50 milliseconds, *PM*_*2.5*_ particulate matter ≤ 2.5 μm in aerodynamic diameter, *RMSSD* root mean square of successive differences, *SDNN* standard deviation of normal-to-normal. *p-value < 0.05; **p-value < 0.01

**Fig. 5:**
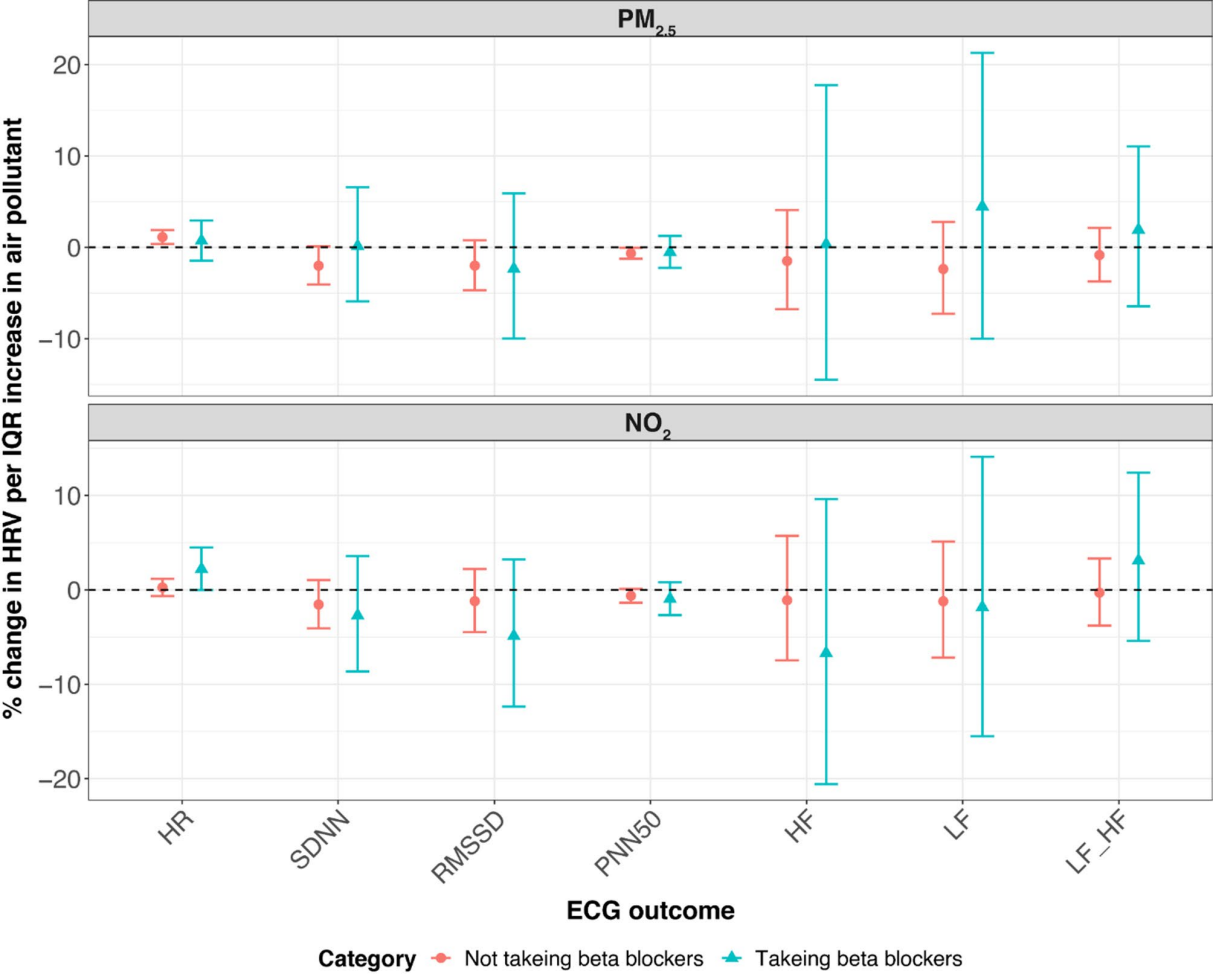
Percent change (95% CI) of the geometric mean of ECG parameters per IQR increase in PM_2.5_ and NO_2_ modified by beta-blockers in KORA S4 at lag M7. *CI* confidence interval, *HF* high frequency power (0.15–0.40 Hz), *HR* heart rate, *IQR* interquartile range, *LF* low frequency power (0.04–0.15 Hz), *LF_HF* low frequency to high frequency ratio, *M2* 2-day moving average, *M5* 5-day moving average, *M7* 7-day moving average, *M14* 14-day moving average, *NO*_*2*_ nitrogen dioxide, *pNN50* percentage of NN intervals longer than 50 milliseconds, *PM*_*2.5*_ particulate matter ≤ 2.5 μm in aerodynamic diameter, *RMSSD* root mean square of successive differences, *SDNN* standard deviation of normal-to-normal beats. *p-value < 0.05; **p-value < 0.01

In KORA S4, we observed significant effect modifications by age, smoking status, and beta-blocker use. To provide a clear summary, we present the results for lag M7 in the main text (Figs. 3, 4 and 5), which are representative of the overall modification patterns. Results for all lag days are shown in the Supplementary Figures S2-S4. Specifically, the positive association between NO₂ and LF/HF ratio was more pronounced in younger individuals. By contrast, the negative association between NO_2_ and LF and the LF/HF ratio was stronger in older participants. Additionally, we found that individuals taking beta-blockers exhibited a stronger positive association between NO_2_ and HR and a more pronounced negative association between NO_2_ and RMSSD. Among non-smokers, there was also a stronger negative association between NO_2_ and HR. Effect modifications by sex, obesity, alcohol consumption, educational attainment, physical activity, occupation, season, CVD, and diabetes were neither significant nor consistent in S4.

In S4, we also conducted a subgroup analysis by grouping participants based on age and smoking status. A total of 344 participants were identified as former or current smokers aged ≥ 65 years, while 357 participants were aged ≥ 65 years and had never smoked. Among those aged < 65 years, 2025 participants were former or current smokers, and 1306 participants had never smoked (Table S3). Overall, the analysis stratified by smoking status and age showed that the absolute values of percent change were generally smaller in the group of older individuals who were former or current smokers (Figure S5).

### Sensitivity analyses

Overall, the associations between short-term exposure to air pollutants and electrocardiographic indices related to rate and rhythm were robust across a series of sensitivity analyses in both S4 and FF4. For example, we observed similar associations when using two-pollutant models; the effects of PM_2.5_ on HR and HRV variables were stronger after adjusting for NO_2_. Furthermore, restricting the S4 analysis to participants present in both S4 and FF4, excluding participants taking beta-blockers, or excluding participants with CVD did not change the results substantially. Excluding outliers in the ECG data did not change the effects (data not shown). The results from the longitudinal sensitivity analysis using IPW were consistent with those from the main longitudinal analysis based on participants with two measurements (Figure S2).

## Discussion

This study examined the effects of short-term exposure to ambient air pollution on HR and HRV across the KORA S4 and FF4 study waves.

In the earlier wave S4, both PM_2.5_ and NO_2_ were associated with increased HR and decreased SDNN (only with PM_2.5_) and pNN50. The cumulative effects over different time frames of up to 14 days of PM_2.5_ and NO_2_ on HR, SDNN, and pNN50 were particularly pronounced, supporting our hypothesis that short-term exposure to air pollution contributes to delayed dysregulation of the ANS, with stronger cumulative effects over time.

However, in the later wave FF4, we observed opposite associations between air pollution and HR and HRV compared to the S4 wave. An animal study suggested that the effects of air pollution exposure on ANS activity may depend on both exposure concentration and duration [34]. Specifically, acute low-concentration exposure to particulate matter initially induced oxidative stress, promoting sympathetic dominance and a reduction in HRV. However, with prolonged exposure or at higher concentrations, pulmonary and airway irritant reflexes appeared to counteract sympathetic activation, resulting in parasympathetic predominance and increased HRV. In the context of our study, the FF4 wave represents a follow-up about 14 years after S4, implying prolonged exposure to PM_2.5_ and NO_2_, which may have contributed to the shift toward parasympathetic predominance observed in FF4. At the same time, compared to those lost to follow-up, participants who remained in the FF4 wave were relatively younger and healthier (Table S2), with lower smoking prevalence, lower BMI, and other favorable characteristics, potentially indicating greater antioxidant defense capacity and reduced oxidative stress in response to pollution. The effect modification results from S4 showed that older participants had lower LF/HF, indicating a stronger parasympathetic response (Figure S3). Taken together, the opposite associations observed in FF4 may partly reflect the older age of participants at the 14-year follow-up and the potential influence of age-related changes in autonomic regulation. The FF4 wave represents a 14-year follow-up from S4, and participants in FF4 were, on average, nearly 14 years older. For those who participated in both S4 and FF4, we also found that their average pack years of smoking increased from 9.14 in S4 to 11.2 in FF4 (Supplement Table S1). To further explore how age and smoking habits might influence the effects of air pollution on ECG parameters, we conducted a stratified analysis based on age and smoking status in model 3. Several of these interaction terms were significant across different outcomes and lag periods. Notably, our subgroup analysis of older participants who were former or current smokers in S4 indicated that this group exhibited smaller absolute values of percent change. This suggests that the impact of air pollution on this subgroup was attenuated. Such findings may, to some extent, explain the discrepancies between the results observed in FF4 and S4, as the lower magnitude of response in older smokers could contribute to the observed inconsistencies.

While most studies have focused on PM_2.5_, relatively few have examined the short-term impact of both PM_2.5_ and NO_2_. Moreover, the findings across studies have been inconsistent, with some showing decreases in time-domain (SDNN and RMSSD) and frequency-domain (HF, LF, and LF/HF ratio) [35–37] indices of HRV and others reporting increases in both time- and frequency-domains indices [38, 39], or no associations at all [39–41].

Our results demonstrated particularly delayed positive associations between PM_2.5_, NO_2_, and HR. The size of the effect estimate increased from lag1 to lag4, with the most substantial estimate (1.47% increase for PM_2.5_ and 1.20% increase for NO_2_) observed at the 14-day moving average. This aligns with previous evidence suggesting that air pollution affects the ANS through delayed mechanisms [14, 42–44].

Although non-significant, in S4, we observed negative associations between PM_2.5_ and NO_2_ and indices of HRV, such as LF, HF, and RMSSD. These findings align with the APACR study from the U.S., which reported significant decreases in LF and HF following short-term exposure to PM_2.5_ with several hours’ lag and no association with RMSSD, but with a decreased trend [45]. Similarly, a longitudinal study from Harvard University found that PM_2.5_ exposure led to a 10.10% decrease in RMSSD among elderly subjects [46], corroborating our findings of delayed and cumulative effects on HRV, even though the results in our study did not reach significance. These findings suggest that PM_2.5_ and NO_2_ exposure may impair parasympathetic function, as indicated by reductions in RMSSD, a key marker of parasympathetic nervous system activity [47, 48].

Furthermore, while many studies have reported negative associations between PM_2.5_ and pNN50 [46, 49, 50], evidence regarding the effects of PM_2.5_ and NO_2_ on pNN50 remains limited. Consistent with findings on PM_2.5_, our study identified a significant negative association between pNN50 and both PM_2.5_ and NO_2_ at 7-day and 14-day moving averages. A reduction in pNN50 reflects decreased parasympathetic nervous system activity and serves as an important marker of impaired autonomic regulation [48, 51, 52].

Many cross-sectional and longitudinal studies observed a negative association between PM_2.5_ and SDNN; evidence for NO_2_ is limited. A cross-over study of non-smoking, healthy women in Canada found that each IQR increase in NO_2_ was associated with a 5.92 ms decrease in SDNN [36]. Our study observed the most significant cumulative effect at a 14-day moving average, indicating that air pollution had a delayed and cumulative influence on SDNN. A reduction in SDNN is consistently associated with increased sympathetic nervous system activity and diminished parasympathetic nervous system activity.

Interestingly, our study observed a positive association between air pollution and HRV, alongside a negative association with HR, particularly in older individuals who smoked. This group may exhibit heightened sensitivity to pollutant exposure due to preexisting respiratory or cardiovascular conditions, leading to HRV responses that differ from those seen in the general population. However, to the best of our knowledge, no previous studies have specifically examined the effects of air pollution on HRV in older adults who smoke. Some studies have reported increased HRV and decreased HR with rising air pollutant levels, particularly among healthy individuals [13, 53]. For example, Stein and colleagues [54] found that HR decreases with every 5-year increase in age. This age-related decline in HR aligns with our findings, reinforcing the understanding that HR tends to decrease as individuals grow older. Riediker et al. [55] similarly observed an increase in HRV among young, healthy highway patrolmen exposed to PM_2.5_, attributing it to enhanced vagal activity. While older smokers may have compromised cardiovascular or respiratory health compared to healthy individuals, these findings suggest that under certain conditions, exposure to pollutants may lead to increased HRV, even in populations with existing health vulnerabilities. A longitudinal study on individuals with chronic obstructive pulmonary disease (COPD) found an increase in SDNN among those with poor pulmonary function, but a decrease in individuals with normal lung function [56]. This indicates that respiratory health may be an important modifier of the autonomic response to pollution exposure, which could explain the contrasting HRV results in our older smoking group. The well-established link between long-term smoking, reduced lung function, and the risk of development of COPD and lung cancer [57–59] further supports the hypothesis that HRV differences in smokers may reflect underlying respiratory health disparities.

In our FF4 study, a higher proportion of individuals reported taking beta-blockers than the S4 cohort, which may also explain the opposite trends observed in HRV between the two studies. Beta-blockers are known to enhance vagal modulation, thereby increasing HRV, particularly in individuals with cardiovascular conditions [60–63]. For example, a randomized, placebo-controlled cross-over study demonstrated that beta-blockers significantly lowered HR while increasing HRV compared to a placebo [60]. This increase in HRV is attributed to heightened parasympathetic activity, which suppresses excessive sympathetic activation, leading to a more stable heart rhythm.

Additionally, in the FF4 wave, conducted 14 years after S4 as a follow-up, the average air pollution levels were lower than S4. Changes in overall air quality over time may have contributed to the differences in HRV responses observed between the two waves.

Of particular relevance is the effect of lower concentrations of air pollution on cardiovascular responses. A recent study by the Max-Planck Institute in Mainz [64] suggests that at low concentrations, oxygen radicals may be generated, potentially activating highly responsive systems like the autonomic nervous system. This activation might lead to beneficial short-term effects, especially in vulnerable populations. However, this interpretation is speculative and requires further research to understand the physiological mechanisms involved. The relatively lower pollution levels in the FF4 cohort compared to S4 might explain some of the contrasting HRV responses observed between the two cohorts, with the differences potentially reflecting changes in air quality and individual health status over time.

We found that short-term exposure to air pollutants has a delayed effect on increasing HR and decreasing HRV in the general population. PM_2.5_ and NO_2_ can penetrate the lungs and trigger biochemical reactions that lead to the production of reactive oxygen species, initiating inflammatory responses [65]. This oxidative stress can stimulate the ANS, particularly the sympathetic nervous system, which releases hormones like adrenaline and norepinephrine. These hormones directly affect the heart, resulting in an increase in HR [66]. Following exposure to air pollution, the body produces an inflammatory response that releases markers such as interleukin-6 and, subsequently, C-reactive protein. These inflammatory responses do not subside quickly but can persist for days or even lead to chronic inflammation [67]. Prolonged inflammation induces sustained cardiovascular stress, keeping HR elevated for several days after the initial exposure [68]. Elevated levels of these inflammatory markers have been associated with increased HR [69]. Additionally, the prolonged presence of free radicals and reactive oxygen species caused by oxidative stress can continue to damage cells [70], exacerbating inflammation and particularly affecting the heart and blood vessels. This sustained state of stress may explain both the lagged and cumulative effects of air pollution on cardiovascular health [71].

NO_2_ may also directly affect cardiovascular function by altering endothelial function and reducing the bioavailability of nitric oxide (NO), which can lead to vasoconstriction, elevated blood pressure, and a subsequent reduction in HRV [72]. Additionally, NO_2_ may affect calcium and other ion channels, thereby impacting the function of the cardiac conduction system and, in turn, further influencing HRV. However, the precise mechanisms through which NO_2_ induces cardiovascular diseases require more in-depth investigation [73].

HRV is not solely influenced by environmental exposures and lifestyle factors such as smoking and physical activity. Aging itself is a significant contributor, as it is associated with intrinsic physiological changes. Research has shown that heavy smokers tend to exhibit higher levels of sympathetic nervous system activation, which can trigger physiological responses, including temporary increases in HRV during certain external stimuli, such as exercise [74]. A study from Switzerland [75] found that regular, healthy smokers had higher HRV indices (LF and LF/HF) and a lower HR compared to healthy non-smokers, which aligns with previous results by Eryonucu and colleagues. Furthermore, a randomized controlled trial (RCT) [76] demonstrated that regular physical activity increased HRV in older subjects, indicating that lifestyle factors such as physical activity can influence HRV changes. As individuals age, not only lifestyle factors but also biological changes play crucial roles in HR and HRV regulation. Numerous studies have documented age-related changes in vagus nerve function, as measured by HRV [77–79]. These studies suggest that HRV decreases after post-pubertal development and may increase again in older adulthood [80]. These findings suggest that a comprehensive understanding of HRV changes requires consideration of environmental factors, lifestyle habits, and the natural physiological changes that occur with aging. Overall, the evidence for air pollution-related changes in HRV is mixed, and these effects are likely influenced by various factors, such as age, lifestyle (e.g., physical activity and smoking status), and other health conditions.

Our study has several strengths. One of the strengths is its large sample size and the extensive number of ECG recordings, making it one of the largest cohort studies to analyze the effects of PM_2.5_ and NO_2_ on HRV indices. Secondly, this study is the combined use of cross-sectional and longitudinal analyses, providing a more comprehensive assessment of air pollution’s impact on cardiovascular autonomic function across distinct time points and over time, providing valuable information on its potential health effects at different stages in life. The diverse population sampled across groups differing in age and health status further enhances the generalizability of the results. Thirdly, by examining HRV indices such as pNN50 and the LF/HF ratio-parameters rarely studied in relation to air pollution - this study offers a more comprehensive analysis of HR and HRV variables in investigating the short-term adverse health effects of ambient air pollution. Lastly, this study provides novel insights into the short-term effects of air pollution on HRV among older smokers; to our knowledge, no prior study has examined this specific population.

However, several limitations of this study should be acknowledged. Firstly, exposure assessments were based on daily urban background rather than personal exposure measurements, which may have led to non-differential exposure misclassification and potentially biased our results toward the null [81]. Secondly, attrition between surveys may have resulted in a healthier remaining sample, potentially biasing the observed associations toward the null. Thirdly, the significant reduction in PM_2.5_ and NO_2_ concentrations over time may have reduced our ability to discern biological effects from the background. Fourthly, the generalizability of our findings to other populations with different ethnic, climatic, or demographic characteristics may be limited, as our observations stem from a single-center cohort study conducted in Augsburg, Germany. Furthermore, due to data limitations, the study did not examine associations between HRV and a broader range of air pollutants.

## Conclusions

In conclusion, this study indicates that short-term exposure to PM_2.5_ and NO_2_ impairs cardiac autonomic function, as reflected by reduced HRV and increased HR. These associations are biologically plausible, since air pollution can induce systemic inflammation and oxidative stress, well-established pathways contributing to autonomic imbalance and elevated cardiovascular risk. The findings were observed in a setting with relatively low ambient concentrations, suggesting that even low-level exposures may influence autonomic regulation. In areas with higher pollution levels and prolonged exposure, the adverse effects are likely to be more pronounced and may substantially increase the population burden of cardiovascular disease. Among older individuals who smoke, smaller absolute changes were observed compared to other groups, indicating a potentially distinct physiological response to pollution. From a public health perspective, the results highlight the importance of reducing air pollution exposure while also considering lifestyle and age-related factors, as such an integrated approach may strengthen cardiovascular risk assessment and guide targeted prevention strategies.

## Supplementary Information

Supplementary Material 1

## Data Availability

No datasets were generated or analysed during the current study.

## Abbreviations

ANS: Autonomic nervous system
BMI: Body mass index
CI: Confidence interval
COPD: Chronic obstructive pulmonary disease
CVD: Cardiovascular disease
DAL Square: Dalhousie University Square
exp: Exposure
F4: First follow-up examination of KORA S4
FF4: Second follow-up examination of KORA S4
GAMs: Generalized additive models
GAMMs: Generalized additive mixed models
GM: Geometric mean
HF: High frequency
HR: Heart rate
HRV: Heart rate variability
IQR: interquartile range
IPW: Inverse probability weighting
KORA: Cooperative Health Research in the Region of Augsburg
LF: Low frequency
LF_HF: Low frequency to high frequency ratio
M2: 2-Day moving average
M5: 5-Day moving average
M7: 7-Day moving average
M14: 14-Day moving average
MI: Myocardial infarction
NO: Nitric oxide
NO_2_: Nitrogen dioxide
pNN50: The percentage of successive RR intervals differing by more than 50 ms
PM: Particulate matter
PM_2.5_: Fine particulate matter
RCT: Randomized controlled trial
RH: Relative humidity
RMSSD: The root mean square of successive RR interval differences
S4: Fourth cross-sectional health survey of the KORA cohort
SBP: Systolic blood pressure
SD: Standard deviations
SDNN: Standard deviation of normal-to-normal intervals
SZ: Sulfur dioxide
T: Temperature
WHO: World Health Organization
