# Supplementary material for: Short-term association between ambient air pollution and heart rate variability: results from the population-based KORA S4 and FF4 studies
Source: Part Fibre Toxicol. 2025 Oct 17;22:26. doi: 10.1186/s12989-025-00645-6 (PMC12534996; doi:10.1186/s12989-025-00645-6)
Supplement: Supplementary file 1 — Supplementary Material 1 [file 12989_2025_645_MOESM1_ESM.docx]

**Short-term association between ambient air pollution and heart rate variability: results from the population-based KORA S4 and FF4 studies**

**Authors:** Yujiao Li^1,2^, Susanne Breitner-Busch^1,2^, Wayne E. Cascio^3^, Siqi Zhang^4^, Kathrin Wolf^1^, Ina-Maria Rückert-Eheberg^1^, Stefan Kääb^5,6^, Georg Schmidt^5,7^, Alexander Strom^8,9^, Annette Peters^1,2,5,9^, Alexandra Schneider^1^

1 Institute of Epidemiology, Helmholtz Zentrum München, German Research Center for Environmental Health (GmbH), Neuherberg, Germany

2 Institute for Medical Information Processing, Biometry and Epidemiology (IBE), Faculty of Medicine, LMU Munich, Pettenkofer School of Public Health, Munich, Germany

3 US Environmental Protection Agency, Durham, North Carolina

4 Department of Environmental Health Sciences, Yale School of Public Health, New Haven, CT, USA

5 German Research Center for Cardiovascular Disease (DZHK), Partner site Munich Heart Alliance, Munich, Germany

6 Department of Cardiology, Medical Policlinic and University Clinic I, Munich, Germany

7 Department of Internal Medicine I, TUM University Hospital, Technical University of Munich, Ismaninger Str. 22, 81675, Munich, Germany

8 Institute for Clinical Diabetology, German Diabetes Center, Leibniz Center for Diabetes Research at Heinrich Heine University Düsseldorf, Düsseldorf, Germany

9 German Center for Diabetes Research (DZD), München-Neuherberg, Germany

**Corresponding author:**

Yujiao Li

Institute of Epidemiology, Helmholtz Zentrum München, German Research Center for Environmental Health (GmbH), Neuherberg, Germany

Institute for Medical Information Processing, Biometry and Epidemiology (IBE), Faculty of Medicine, LMU Munich, Pettenkofer School of Public Health, Munich, Germany

E-Mail: [yujiao.li@helmholtz-munich.de](mailto:yujiao.li@helmholtz-munich.de)

Pages: 11

Tables: 3

Figures: 6

**List of Tables and Figures**

**Table S1. Descriptive statistics of baseline and follow-up participant characteristics for individuals participating in both KORA S4 and FF4.**

**Table S2. Baseline characteristics of KORA S4 participants with and without follow-up data in FF4.**

**Table S3. Sample size stratified by age and smoking status in S4.**

**Figure S1. Percent change (95% CI) of the geometric mean of ECG parameters per interquartile range increase in PM_2.5_ and NO_2_, based on the longitudinal analysis of participants involved in both KORA S4 and FF4.**

**Figure S2. Percent change (95% CI) of the geometric mean of ECG parameters per interquartile range increase in PM_2.5_ and NO_2_, based on the longitudinal analysis with IPW in KORA S4 and FF4.**

**Figure S3. Percent change (95% CI) of the geometric mean of ECG parameters per IQR increase in PM_2.5_ and NO_2_ modified by age in KORA S4.**

**Figure S4. Percent change (95% CI) of the geometric mean of ECG parameters per IQR increase in PM_2.5_ and NO_2_ modified by smoking in KORA S4.**

**Figure S5. Percent change (95% CI) of the geometric mean of ECG parameters per IQR increase in PM_2.5_ and NO_2_ modified by beta-blockers in KORA S4.**

**Figure S6. Percent change (95% CI) of the geometric mean of ECG parameters per IQR increase in PM_2.5_ and NO_2_ stratified by age and smoking status in KORA S4.**

**Table S1.** Descriptive statistics of baseline and follow-up participant characteristics for individuals participating in both KORA S4 and FF4.

|  | **Mean (SD) or number (%)** | | ***P* value*** |
| --- | --- | --- | --- |
|  | **S4 (N = 1,843)** | **FF4 (N = 1,843)** |  |
| **Age (years)** | 45.1 (11.7) | 58.7 (11.7) | <0.001 |
| **Sex (male)** | 852 (46.2) | 852 (46.2) | 1.000 |
| **BMI (kg/m^2^)** | 26.5 (4.3) | 27.6 (4.9) | 0.001 |
| **Obesity (% yes)** | 332 (18.0) | 494 (26.8) | 0.003 |
| **Smoking status (% yes)** |  |  | <0.001 |
| Current smoker | 448 (24.3) | 302 (16.4) |  |
| Former smoker | 620 (33.6) | 772 (41.9) |  |
| Never smoker | 775 (42.1) | 769 (41.7) |  |
| **Smoking Pack years (years)** | 9.14 (16.0) | 11.2 (18.7) | <0.001 |
| **Alcohol status (% yes)** | 1398 (75.9) | 1342 (72.8) | 0.038 |
| **Physical activity*** |  |  | 0.010 |
| Low | 524 (28.4) | 493 (26.7) |  |
| Medium | 913 (49.5) | 865 (46.9) |  |
| High | 406 (22.0) | 485 (26.3) |  |
| **Educational attainment** |  |  | 1.000 |
| College | 477 (25.9) | 477 (25.9) |  |
| High school | 498 (27.0) | 498 (27.0) |  |
| Primary school | 868 (47.1) | 868 (47.1) |  |
| **Occupational status (% yes)** | 1252 (67.9) | 1004 (54.5) | <0.001 |
| **Diabetes (% yes)** | 34 (1.8) | 160 (8.7) | <0.001 |
| **MI (% yes)** | 16 (0.9) | 48 (2.6) | <0.001 |
| **Stroke (% yes)** | 3 (0.2) | 33 (1.8) | <0.001 |
| **Hypertension (% yes)** | 522 (28.3) | 661 (35.9) | <0.001 |
| **CVD (% yes)** | 528 (28.6) | 672 (36.5) | <0.001 |
| **Intake of beta blocker (% yes)** | 113 (6.1) | 325 (17.6) | <0.001 |
| **Intake of medication (% yes)** | 324 (17.6) | 681 (37.0) | <0.001 |
| **Season** |  |  | <0.001 |
| spring | 636 (34.5) | 398 (21.6) |  |
| summer | 343 (18.6) | 591 (32.1) |  |
| autumn | 275 (14.9) | 503 (27.3) |  |
| winter | 589 (32.0) | 351 (19.0) |  |
| **Season (% cold)** | 1225 (66.5) | 854 (46.3) | <0.001 |

* *P* value was estimated by t-test (continuous variables) or Chi-squared test (categorical variables). *Physical activity* was categorized based on the time spent on physical exercise into low (no or almost no physical exercise), medium (about one hour per week), and high (more than two hours per week).

Abbreviations: BMI, body mass index; CVD, participant has hypertension or stroke or MI; FF4, second follow-up examination of KORA S4; KORA, Cooperative Health Research in the Region of Augsburg; MI, myocardial infarction; S4, fourth cross-sectional health survey of the KORA cohort; SD, standard deviation.

**Table S2.** Baseline characteristics of KORA S4 participants with and without follow-up data in FF4.

|  | **Mean (SD) or number (%)** | | ***P* value*** |
| --- | --- | --- | --- |
|  | **S4 with follow-up data (N = 1,843)** | **S4 without follow-up data (N = 2,189)** |  |
| **Age (years)** | 45.1 (11.7) | 52.4 (14.6) | <0.001 |
| **Sex (male)** | 852 (46.2) | 1122 (51.3) | 0.002 |
| **BMI (kg/m^2^)** | 26.5 (4.3) | 27.7 (5.0) | <0.001 |
| **Obesity (% yes)** | 332 (18.0) | 607 (27.7) | <0.001 |
| **Smoking status (% yes)** |  |  | 0.081 |
| Current smoker | 448 (24.3) | 600 (27.4) |  |
| Former smoker | 620 (33.6) | 701 (32.0) |  |
| Never smoker | 775 (42.1) | 888 (40.6) |  |
| **Smoking Pack years (years)** | 9.14 (16.0) | 12.3 (19.3) | <0.001 |
| **Alcohol status (% yes)** | 1398 (75.9) | 1533 (70.0) | <0.001 |
| **Physical activity*** |  |  | <0.001 |
| Low | 524 (28.4) | 840 (38.4) |  |
| Medium | 913 (49.5) | 931 (42.5) |  |
| High | 406 (22.0) | 418 (19.1) |  |
| **Educational attainment** |  |  | <0.001 |
| College | 477 (25.9) | 442 (20.2) |  |
| High school | 498 (27.0) | 451 (20.6) |  |
| Primary school | 868 (47.1) | 1296 (59.2) |  |
| **Occupational status (% yes)** | 1252 (67.9) | 1010 (46.1) | <0.001 |
| **Diabetes (% yes)** | 34 (1.8) | 119 (5.4) | <0.001 |
| **MI (% yes)** | 16 (0.9) | 64 (2.9) | <0.001 |
| **Stroke (% yes)** | 3 (0.2) | 45 (2.1) | <0.001 |
| **Hypertension (% yes)** | 522 (28.3) | 954 (43.6) | <0.001 |
| **CVD (% yes)** | 528 (28.6) | 973 (44.4) | <0.001 |
| **Intake of beta blocker (% yes)** | 113 (6.1) | 313 (14.3) | <0.001 |
| **Intake of medication (% yes)** | 324 (17.6) | 684 (31.2) | <0.001 |
| **Season** |  |  | 0.052 |
| spring | 636 (34.5) | 845 (38.6) |  |
| summer | 343 (18.6) | 385 (17.6) |  |
| autumn | 275 (14.9) | 320 (14.6) |  |
| winter | 589 (32.0) | 639 (29.2) |  |
| **Season (% cold)** | 1225 (66.5) | 1484 (67.8) | 0.390 |

* *P* value was estimated by t-test (continuous variables) or Chi-squared test (categorical variables). *Physical activity* was categorized based on the time spent on physical exercise into low (no or almost no physical exercise), medium (about one hour per week), and high (more than two hours per week).

Abbreviations: BMI, body mass index; CVD, participant has hypertension or stroke or MI; FF4, second follow-up examination of KORA S4; KORA, Cooperative Health Research in the Region of Augsburg; MI, myocardial infarction; S4, fourth cross-sectional health survey of the KORA cohort; SD, standard deviation.

**Table S3.** Sample size stratified by age and smoking status in S4.

| Age group | Never smokers (N) | Former or current smokers (N) |
| --- | --- | --- |
| Age < 65 | 1306 | 2025 |
| Age ≥ 65 | 357 | 344 |


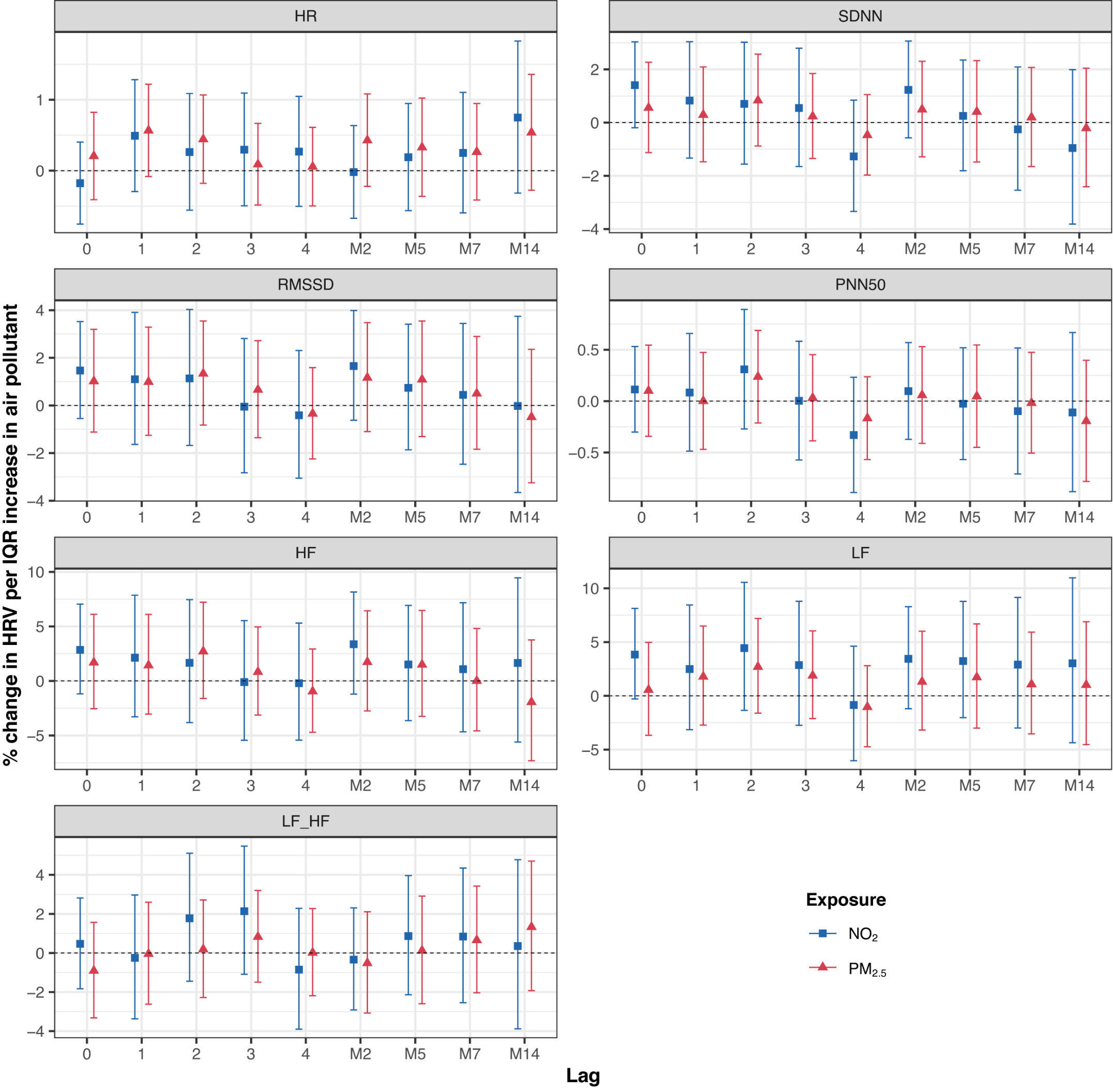


**Figure S1.** Percent change (95% CI) of the geometric mean of ECG parameters per interquartile range increase in PM_2.5_ and NO_2_, based on the longitudinal analysis of participants involved in both KORA S4 and FF4.

Abbreviations: CI, confidence interval; HF, high frequency power (0.15–0.40 Hz); HR, heart rate; IQR, interquartile range; LF, low frequency power (0.04 - 0.15 Hz); LF_HF, low frequency to high frequency ratio; M2, 2-day moving average; M5, 5-day moving average; M7, 7-day moving average; M14, 14-day moving average; NO_2_, nitrogen dioxide; pNN50, percentage of NN intervals longer than 50 milliseconds; PM_2.5_, particulate matter =2.5μm in aerodynamic diameter; RMSSD, root mean square of successive differences; SDNN, standard deviation of normal-to-normal beats.


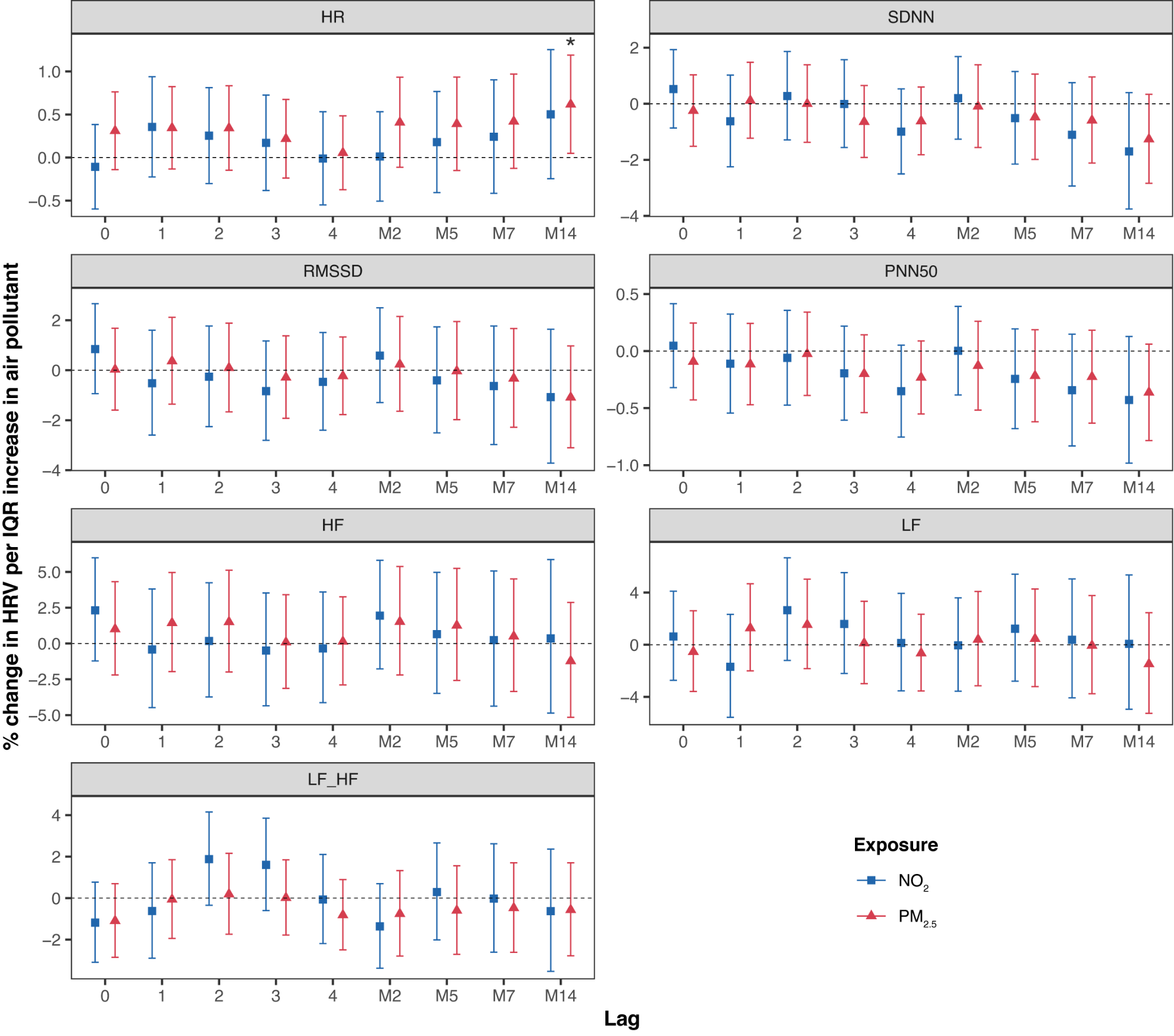


**Figure S2.** Percent change (95% CI) of the geometric mean of ECG parameters per interquartile range increase in PM_2.5_ and NO_2_, based on the longitudinal analysis with IPW in KORA S4 and FF4.

Abbreviations: CI, confidence interval; HF, high frequency power (0.15–0.40 Hz); HR, heart rate; IPW, inverse probability weighting; IQR, interquartile range; LF, low frequency power (0.04 - 0.15 Hz); LF_HF, low frequency to high frequency ratio; M2, 2-day moving average; M5, 5-day moving average; M7, 7-day moving average; M14, 14-day moving average; NO_2_, nitrogen dioxide; pNN50, percentage of NN intervals longer than 50 milliseconds; PM_2.5_, particulate matter =2.5μm in aerodynamic diameter; RMSSD, root mean square of successive differences; SDNN, standard deviation of normal-to-normal beats.


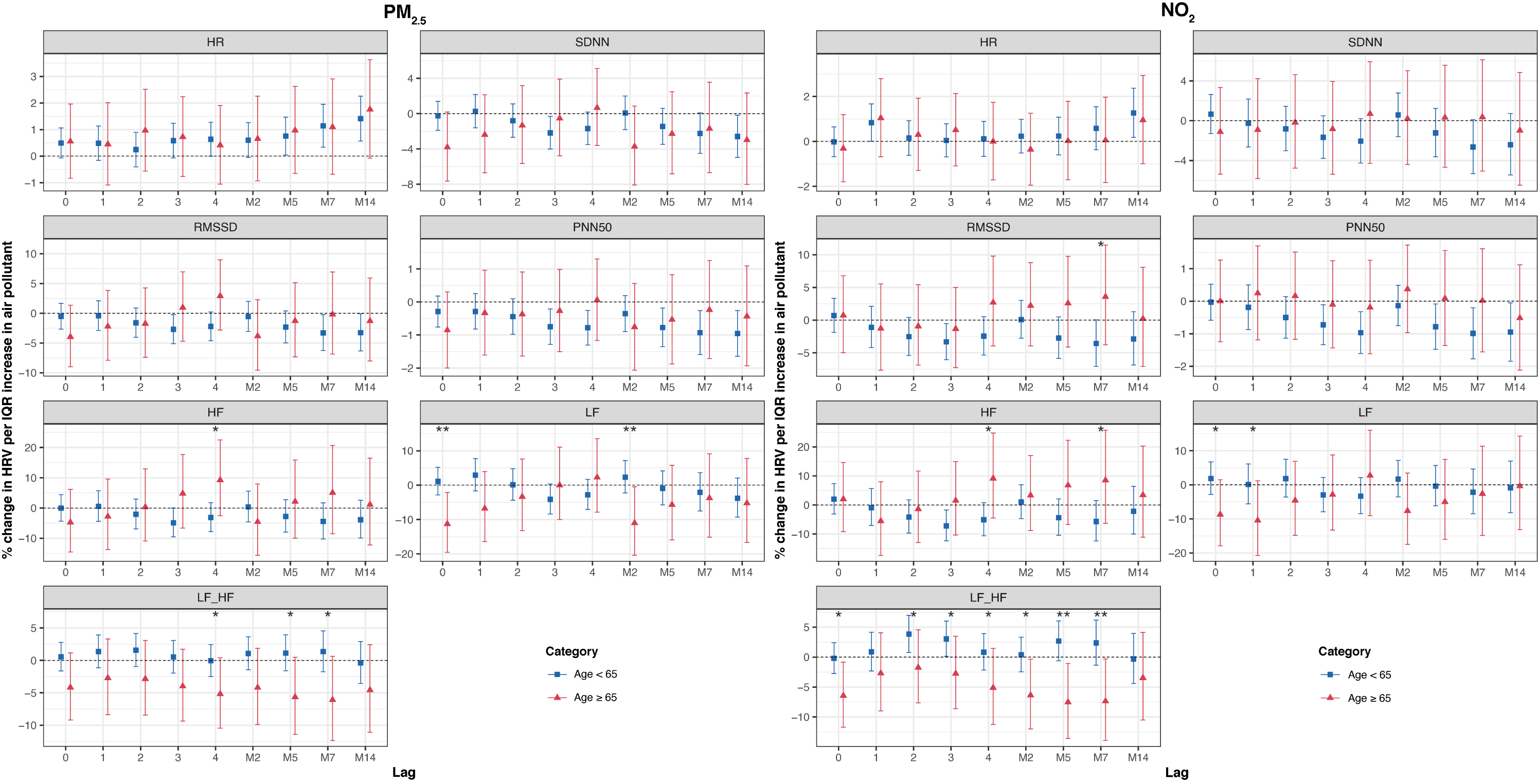


**Figure S3** Percent change (95% CI) of the geometric mean of ECG parameters per IQR increase in PM_2.5_ and NO_2_ modified by age in KORA S4.

Abbreviations: CI, confidence interval; HF, high frequency power (0.15 - 0.40 Hz); HR, heart rate; IQR, interquartile range; LF, low frequency power (0.04 - 0.15 Hz); LF_HF, low frequency to high frequency ratio; M2, 2-day moving average; M5, 5-day moving average; M7, 7-day moving average; M14, 14-day moving average; NO_2_, nitrogen dioxide; pNN50, percentage of NN intervals longer than 50 milliseconds; PM_2.5_, particulate matter = 2.5μm in aerodynamic diameter; RMSSD, root mean square of successive differences; SDNN, standard deviation of normal-to-normal beats.

**p*-Value <0.05; ***p*-Value <0.01


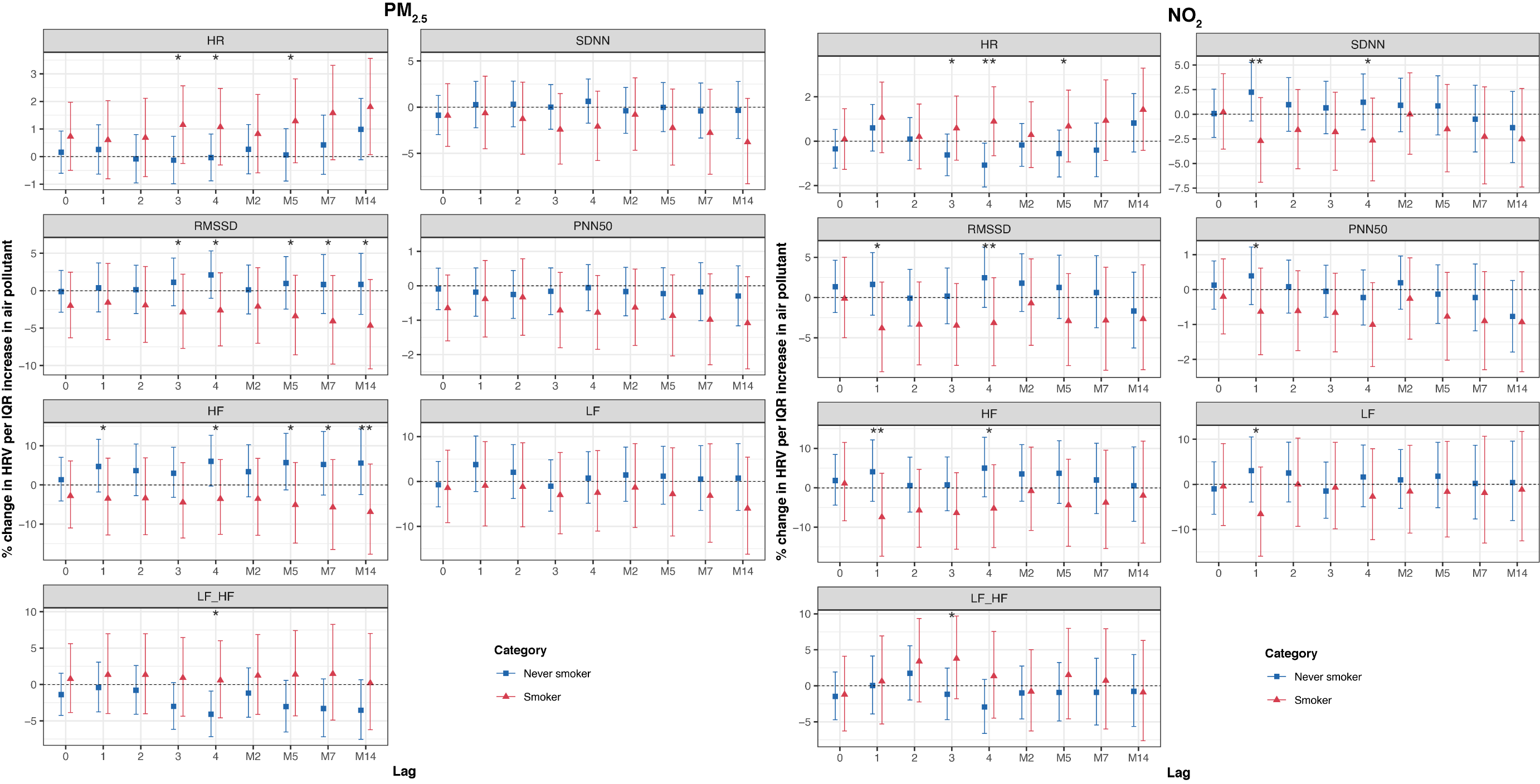


**Figure S4.** Percent change (95% CI) of the geometric mean of ECG parameters per IQR increase in PM_2.5_ and NO_2_ modified by smoking in KORA S4.

Abbreviations: CI, confidence interval; HF, high frequency power (0.15 - 0.40 Hz); HR, heart rate; IQR, interquartile range; LF, low frequency power (0.04 - 0.15 Hz); LF_HF, low frequency to high frequency ratio; M2, 2-day moving average; M5, 5-day moving average; M7, 7-day moving average; M14, 14-day moving average; NO_2_, nitrogen dioxide; pNN50, percentage of NN intervals longer than 50 milliseconds; PM_2.5_, particulate matter = 2.5μm in aerodynamic diameter; RMSSD, root mean square of successive differences; SDNN, standard deviation of normal-to-normal beats.

**p*-Value <0.05; ***p*-Value <0.01


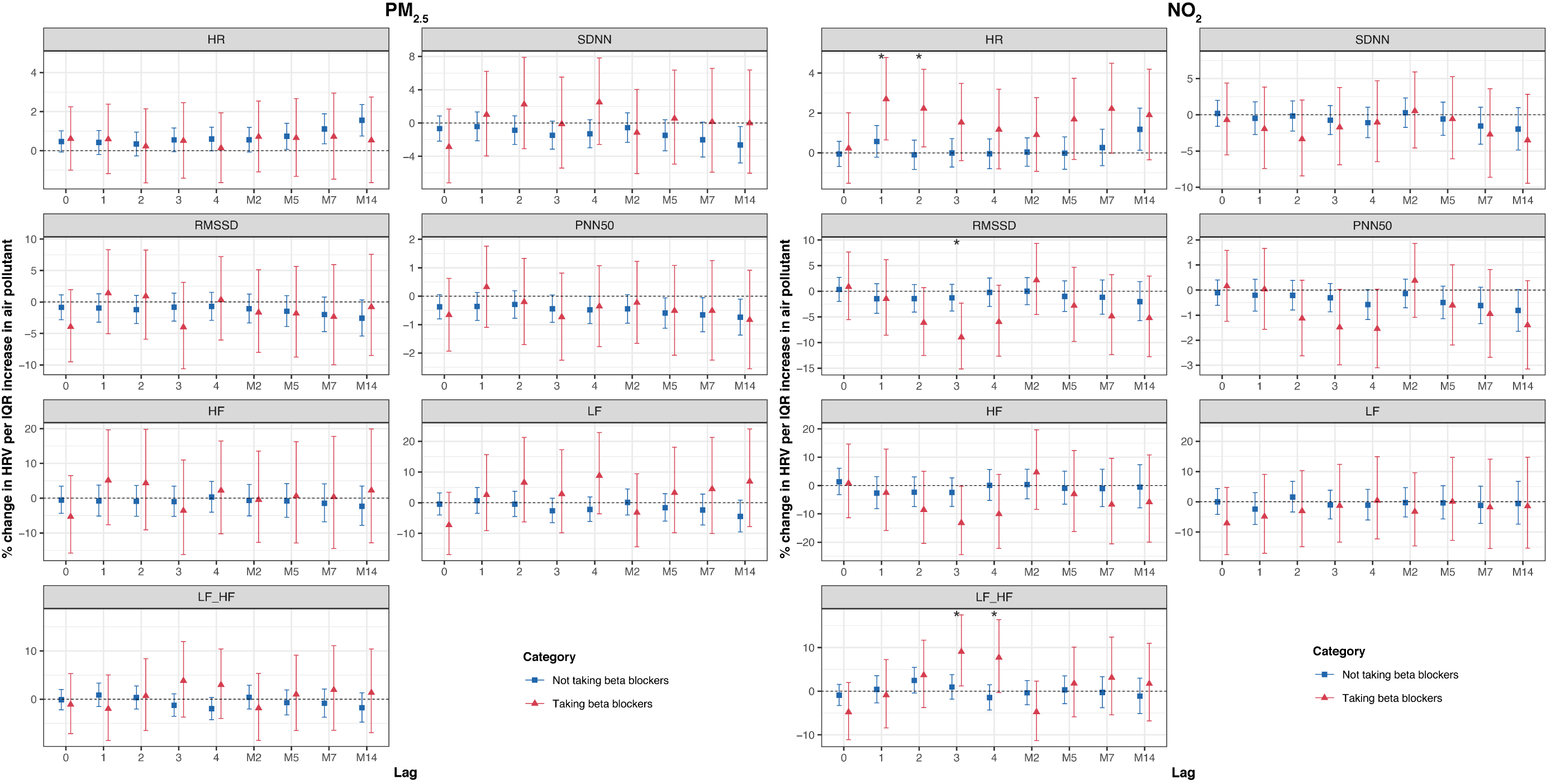


**Figure S5.** Percent change (95% CI) of the geometric mean of ECG parameters per IQR increase in PM_2.5_ and NO_2_ modified by beta-blockers in KORA S4.

Abbreviations: CI, confidence interval; HF, high frequency power (0.15 - 0.40 Hz); HR, heart rate; IQR, interquartile range; LF, low frequency power (0.04 - 0.15 Hz); LF_HF, low frequency to high frequency ratio; M2, 2-day moving average; M5, 5-day moving average; M7, 7-day moving average; M14, 14-day moving average; NO_2_, nitrogen dioxide; pNN50, percentage of NN intervals longer than 50 milliseconds; PM_2.5_, particulate matter = 2.5μm in aerodynamic diameter; RMSSD, root mean square of successive differences; SDNN, standard deviation of normal-to-normal beats.

**p*-Value <0.05; ***p*-Value <0.01


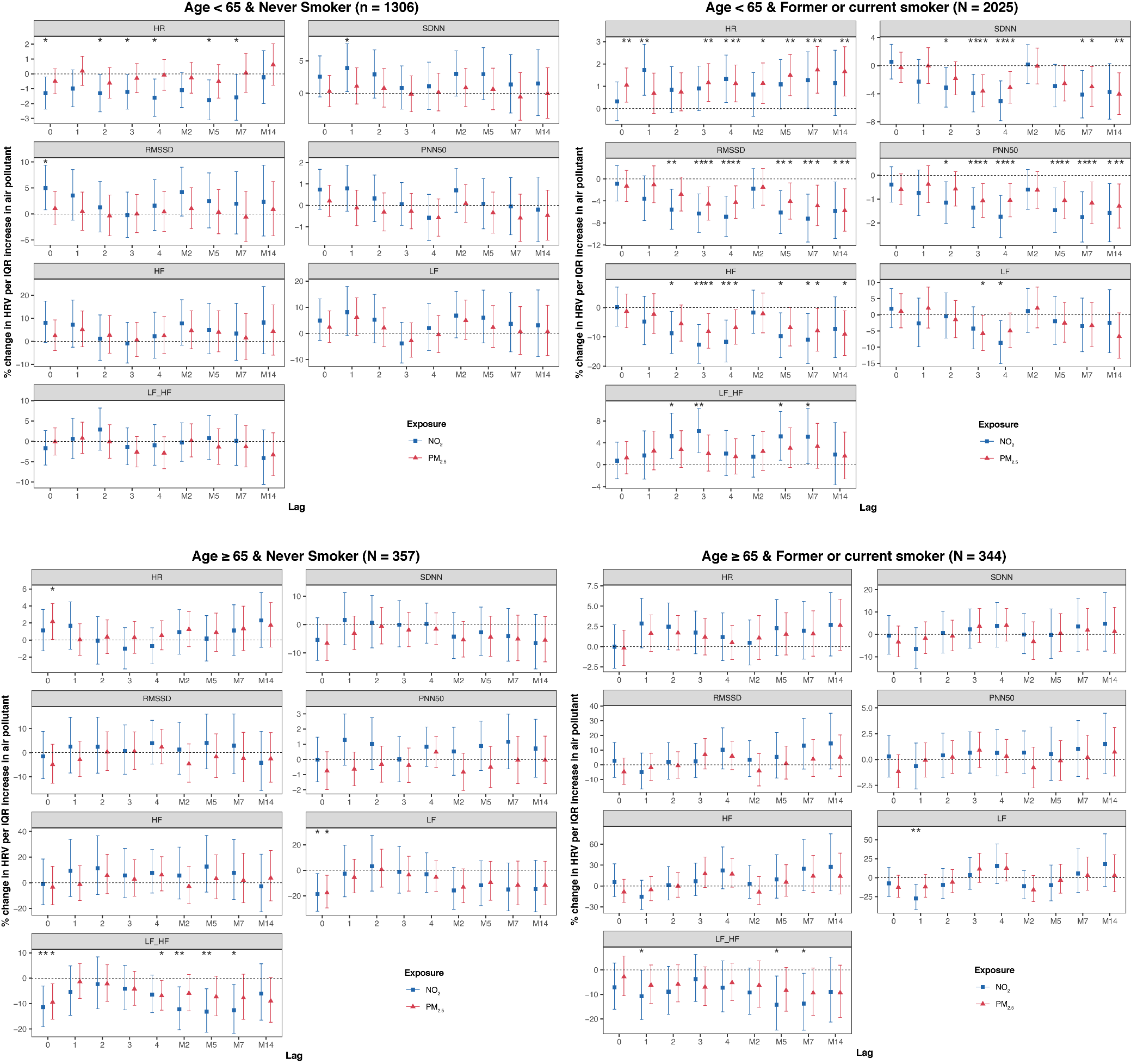


**Figure S6.** Percent change (95% CI) of the geometric mean of ECG parameters per IQR increase in PM_2.5_ and NO_2_ stratified by age and smoking status in KORA S4.

Abbreviations: CI, confidence interval; HF, high frequency power (0.15 - 0.40 Hz); HR, heart rate; IQR, interquartile range; LF, low frequency power (0.04 - 0.15 Hz); LF_HF, low frequency to high frequency ratio; M2, 2-day moving average; M5, 5-day moving average; M7, 7-day moving average; M14, 14-day moving average; NO_2_, nitrogen dioxide; pNN50, percentage of NN intervals longer than 50 milliseconds; PM_2.5_, particulate matter = 2.5μm in aerodynamic diameter; RMSSD, root mean square of successive differences; SDNN, standard deviation of normal-to-normal beats.

**p*-Value <0.05; ***p*-Value <0.01
